# Influence of Christianity on Medical Science

**Published:** 1854-06

**Authors:** John Cumming

**Affiliations:** London


### INFLUENCE OF CHRISTIANITY ON MEDICAL SCIENCE.

(BY REV. JOHN CUMMING OF LONDON.)

EVER since Jesus suffered, wrought miracles, healed the sick, stilled the ocean, and showed his control over rebellious nature —by bringing it back again into order,—man has gained by degrees a greater mastery over all things, as if then humanity received a new impulse ; and in proportion to his Christian light (I do not say Christianity is the cause, but it certainly is a coincidence,) has been his civilization ; and, in proportion to that, the gradual authority which he seems to be regaining over that nature, the reins of which he lost in Paradise, but which Jesus has now partially, and will again completely put into his redeemed and sanctified hand. It is to me a most delightful experience, to see any one discovery in science or in art, which restores to man, however slightly, the mastery over created things. Is it not true that since Jesus healed the sick, there has been given a greater impulse to curative science than ever was felt before ? Is not medicine, with all its defects, with all the obloquy cast upon it, because it cannot do everything, progressive ? Is it not true, that some diseases, once thought incurable, are now almost extirpated ? Small-pox is now, not only curable, but almost banished from our land. And was the discovery of this mode of cure simply chance ? Will you say it was accident ? I believe it to have been as much an inspiration of the God of providence as the bible is an inspiration of the God of grace. Is it not fact, that man's life is longer than it was ? If you do not believe me, ask the Insurance Societies, and they will tell you it is so by some six years. It is much longer than this, if we remember, that the sickly and delicate infant which was lost before, while only the strong ones survived, is now spared, and, under the blessing of God, and by the appliance of art, grows up to manhood. Is not all this gain ? Is it not progress in the direction in which the miracles of Jesus lay, and in the reversal of that curse which "brought death into the world, and all our woe ?" Is it not also true, that operations once thought perfectly impossible, are now performed by our surgeons with safety and success ? Is not that recent wonderful discovery, chloroform, one of the most providential blessings that God has given us? I look upon it as a most significant instalment of the reversal of the curse, stilling the groans and travail of the creature, an inspiration from God; and connected with the special curse pronounced upôn Eve and her daughters, and read in the light of that curse, it is, to my mind, a beautiful earnest of what will be—a forelight of the approaching dawn—an augury of millennial days, when there shall be no more pain, nor tears, nor sorrow, nor crying.

THE MILLENNIAL SABBATH!

It will be a day of lasting rest. When the night that is far spent is completely exhausted, and the day that shall be is fully come, then there shall be perfect rest. The earth shall have his sabbath, which it lost by our sin. Man shall have his, in its integrity, and purity, and beauty. God rested on the seventh day from all his work, and hallowed the sabbath, and blessed it. I believe there is not a beast in the field, nor a fish in the sea nor a fowl in the air, that has not a right to the sabbath, and that shall not yet have a sabbath of rest. There is not a laborer in the workshop, nor a toiling man in the post-office, nor a clerk in the counting-house, that may not claim the sabbath. Next to God's word, God's sabbath is the right and privilege of man. And when that last sabbath comes—the sabbath of all creation—the heart, wearied with its tumultuous beatings, shall have rest; the soul, fevered with its anxieties, shall enjoy peace. The sun of that sabbath will never set, or veil his splendors in a cloud. The flowers that grow in his light will never fade. Our earthly sabbaths are but faint reflections of the heavenly sabbath, cast down upon the earth, dimmed by the transit of their rays from so great a height and so distant a world. The fairest landscapes, or combinations of scenery, upon earth, are but the outskirts of the paradise of God, fore-earnests and intimations of that which lies beyond them; and the happiest sabbath-heart, whose every pulse is a sabbath-bell, hears but a very inadequate echo of the chimes and harmonies of that sabbath, that rest, where we "rest not day and night," in which the song is ever new, and yet ever sung.

